# Synthesis of hafnium nanoparticles and hafnium nanoparticle films by gas condensation and energetic deposition

**DOI:** 10.3762/bjnano.9.179

**Published:** 2018-06-27

**Authors:** Irini Michelakaki, Nikos Boukos, Dimitrios A Dragatogiannis, Spyros Stathopoulos, Costas A Charitidis, Dimitris Tsoukalas

**Affiliations:** 1 Department of Physics, National Technical University of Athens, Heroon Politechniou 9, Zographou - Athens, 15780, Greece; 2 Institute of Nanosciences and Nanotechnology, National Centre for Scientific Research Demokritos, Patriarchou Grigoriou E’ & Neapoleos Str., Aghia Paraskevi - Athens, 15310, Greece; 3 School of Chemical Engineering, National Technical University of Athens, Heroon Politechniou 9, Zographou - Athens, 15780, Greece

**Keywords:** energetic deposition, hafnium, inert-gas condensation, nanomechanical properties, nanoparticles, nanoparticle thin films

## Abstract

In this work we study the fabrication and characterization of hafnium nanoparticles and hafnium nanoparticle thin films. Hafnium nanoparticles were grown in vacuum by magnetron-sputtering inert-gas condensation. The as deposited nanoparticles have a hexagonal close-packed crystal structure, they possess truncated hexagonal biprism shape and are prone to surface oxidation when exposed to ambient air forming core–shell Hf/HfO_2_ structures. Hafnium nanoparticle thin films were formed through energetic nanoparticle deposition. This technique allows for the control of the energy of charged nanoparticles during vacuum deposition. The structural and nanomechanical properties of the nanoparticle thin films were investigated as a function of the kinetic energy of the nanoparticles. The results reveal that by proper adjustment of the nanoparticle energy, hexagonal close-packed porous nanoparticle thin films with good mechanical properties can be formed, without any additional treatment. It is shown that these films can be patterned on the substrate in sub-micrometer dimensions using conventional lithography while their porosity can be well controlled. The fabrication and experimental characterization of hafnium nanoparticles is reported for the first time in the literature.

## Introduction

In the past decades, the interest in and the exploitation of metal nanoparticles (NPs) spread across many areas of nanoscience. Metal NPs and nanoparticles thin films (NTFs) with optimized morphology and structure have attracted significant interest in numerous research areas including catalysis [1–2], sensing [3–4], optics [5–6], data storage [7–8] and biotechnology [9–10]. In many applications the functionality of metal NPs is profoundly affected by their size, shape and structure. For example, NPs with sharp edges reveal enhanced catalytic activity because they provide more active sites for catalytic reactions [11], the “small-size effect” of metal-oxide nanoparticles increases remarkably the sensitivity of gas sensors [12], twinned silver NPs show important different chemical activity compared to their single crystalline counterparts [13]. These and many other examples illustrate that investigating the morphology and structure of metal NPs is essential for their efficient utilization. While for face-centered (fcc) metal NPs a lot of studies have been made [14–16], the knowledge regarding hexagonal close-packed (hcp) metal nanoparticles, despite their interesting properties, is limited. Some examples of hcp nanoparticles reported in bibliography are ruthenium NPs, which demonstrate high catalytic activity during hydrogenation of levulinic acid [17], nickel NPs, which find application as electrochemical sensor [18], and cobalt NPs, which exhibit high magnetic anisotropy [19]. Recently, we have reported that hcp hafnium nanoparticles fabricated by inert-gas condensation, when embedded into a metal-oxide layer, result in a completely new behavior of resistive switching compared with pristine metal-oxide layers, which is important for operation and understanding of nonvolatile resistive random-access memory (ReRAM) [20].

Furthermore except of size, shape and structure of NPs, a crucial issue affecting the performance of NTFs is their porosity. The availability of pores in metallic or metal-oxide NTFs and the tunability of their porosity [21], affects their optical [22] and electrical [23] properties, as well as corresponding applications, which can range from sensors [24] over catalysis [25] to energy storage [26–27]. While an increasing number of applications of porous NTFs are proposed in the literature, the poor mechanical stability of these systems is a major drawback that prevents their widespread industrial use. The weak adhesive force between the nanoparticles leads to fragile coatings that tend to fracture under small loads, making them in many cases unsuitable for industrial use. Mechanical reinforcement is necessary to improve mechanical stability. Some proposed methods in this direction are calcination [28], atomic layer deposition [29], hydrothermal treatment [30] and molecular bonding [31].

Although hafnium and hafnium oxide materials find many applications in various fields, there is a lack of studies on fabrication and structure of hafnium nanoparticles. Hafnium is, for example, used as an impurity in safety systems of nuclear reactors because of its large cross section in neutron absorption [32]. Hafnium alloys are used in medical applications because they are biocompatible and exhibit high corrosion resistance as well as in aerospace technology because it can increase the mechanical strength of materials [33]. Furthermore, because of its high dielectric constant HfO_2_ and its compounds are used in the field of microelectronics for the manufacturing of integrated circuits and more particularly as gate dielectrics of metal-oxide semiconductor transistors having replaced the traditional thermally grown SiO_2_ dielectric [34]. More recently, HfO_2_ was also studied in the same field for non-volatile resistive memory ReRam [35] applications as well as for ferroelectric non-volatile memories [36].

In this context, we present an analytical study on synthesis and structural characterization of Hf NPs and NTFs. Studies on the synthesis and characterization of hcp Hf NPs have been missing and the crystal structure has been only theoretically investigated [37]. In this study, Hf NPs were fabricated by inert-gas condensation (IGC) at room temperature. IGC is a single-step NP synthesis method in a high-vacuum environment. The method allows for a high degree of deposition parameter control. By adjusting the deposition parameters modifications of size, shape and structure of the NPs can be achieved, allowing for the design of NPs according to specific applications [38–39]. In this study, the structure, shape and size of hafnium nanoparticles were characterized by combining X-ray diffraction and transmission electron microscopy (TEM). To our knowledge this is the first report about the fabrication and characterization of Hf NPs. The fabrication of nanoparticles with IGC leads to the charging of a significant fraction of the as formed NPs and, thus, their kinetic landing energy can be controlled via a bias on the substrate. The substrate bias voltage (*V*_s_) enables the NPs either to “soft land” (low-energy deposition) preserving their size and shape, or to land with high-energy impact (high-energy deposition), which depending on the landing energy leads to different morphologies and properties of the NTFs [40]. This method, named here energetic nanoparticle deposition, was first introduced by Haberland two decades ago [41]. Herein, we apply this method in order to produce porous Hf NTFs with good mechanical properties. By controlling the kinetic energy of the nanoparticles upon landing on the substrate, we tune the porosity and the mechanical properties of the porous NTFs. The mechanical properties of the NTFs were investigated by nanoidentation measurements. We demonstrate this method with Hf nanoparticles but it can be applied to any metallic nanoparticles. We propose this single-step method as an alternative solution, to reinforce NTFs and produce mechanical stable porous NTFs. Furthermore we demonstrate this technique to fabricate 3D patterns composed of nanoparticles.

## Results and Discussion

### Soft landing (*V*_s_ = 0 kV) – deposition conditions

Hf NPs were produced by inert-gas condensation. In order to avoid contaminants the base pressure prior to deposition was ca. 9·10^−7^ mbar, a high-purity (99.95%) sputtering Hf target was utilized, and thorough target presputtering was performed prior to deposition. All depositions were performed at room temperature, the argon (Ar) flow rate was in all cases kept at 60 sccm and the sputtering power at 32 W. During deposition in the soft-landing regime, no substrate bias voltage was applied (*V*_s_ = 0 kV). Thus, the kinetic energy of the nanoparticles was mainly defined by the pressure difference between the aggregation and deposition chamber. In this case of low-energy deposition the energy per atom *E*_at_ is about 0.1 eV/atom, which is far below the cohesive energy of the atoms constituting the NPs and nanoparticles do not undergo significant distortion of their shape and size [42–43]. Nanoparticles of different mean dimensions were produced by changing the aggregation-zone length from *D* = 50 mm to *D* = 100 mm. For aggregation-zone lengths below *D* = 50 mm no deposition was recorded. Thus, *D* = 50 mm led to the smallest Hf NP size that could be fabricated. Aggregation-zone lengths above *D* = 100 mm are not supported by the system. Thus, *D* = 100 mm led to the biggest Hf nanoparticle dimensions. No size selection was applied and Hf NPs with a size distribution were fabricated.

### Soft landing – morphological characterization

**Figure 1:**
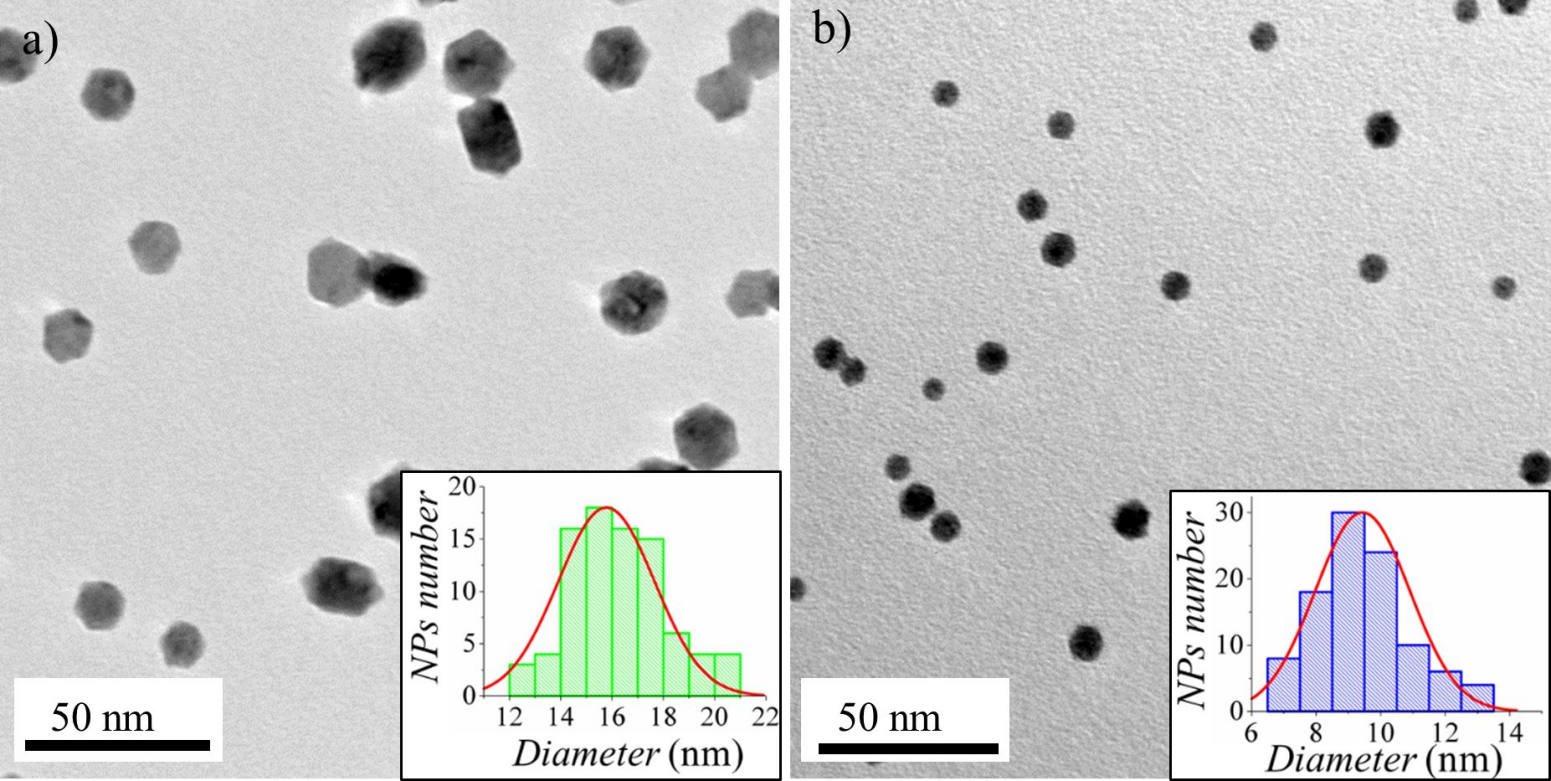
TEM image of the as deposited Hf nanoparticles with mean sizes of (diameter × height) a) 16 nm × 15 nm (aggregation-zone length *D* = 100 mm); b) 9.5 nm × 7 nm (aggregation-zone length *D* = 50 mm). The inset shows a histogram with the diameter distribution. In both cases faceted NPs are formed.

The dimensions (diameter and height) of the Hf NPs were examined by TEM (Figure 1) and AFM. For this purpose samples with low coverage (monolayer) of Hf NPs were prepared. The coverage with nanoparticles was defined by the deposition time. AFM and TEM images reveal the height and diameter of the NPs. Faceted Hf nanoparticles were formed with a mean size of (diameter × height) 16 nm × 15 nm and 9.5 nm × 7 nm for aggregation-zone lengths of *D* = 100 mm and *D* = 50 mm, respectively (Figure 1). All other deposition parameters were identical. The observed increment in size with increasing aggregation-zone length *D* means that the NPs mainly grow in the aggregation zone. The longer residence time of the NPs in the aggregation zone results in the formation of bigger NPs.

### Soft landing – structural characterization

**Figure 2:**
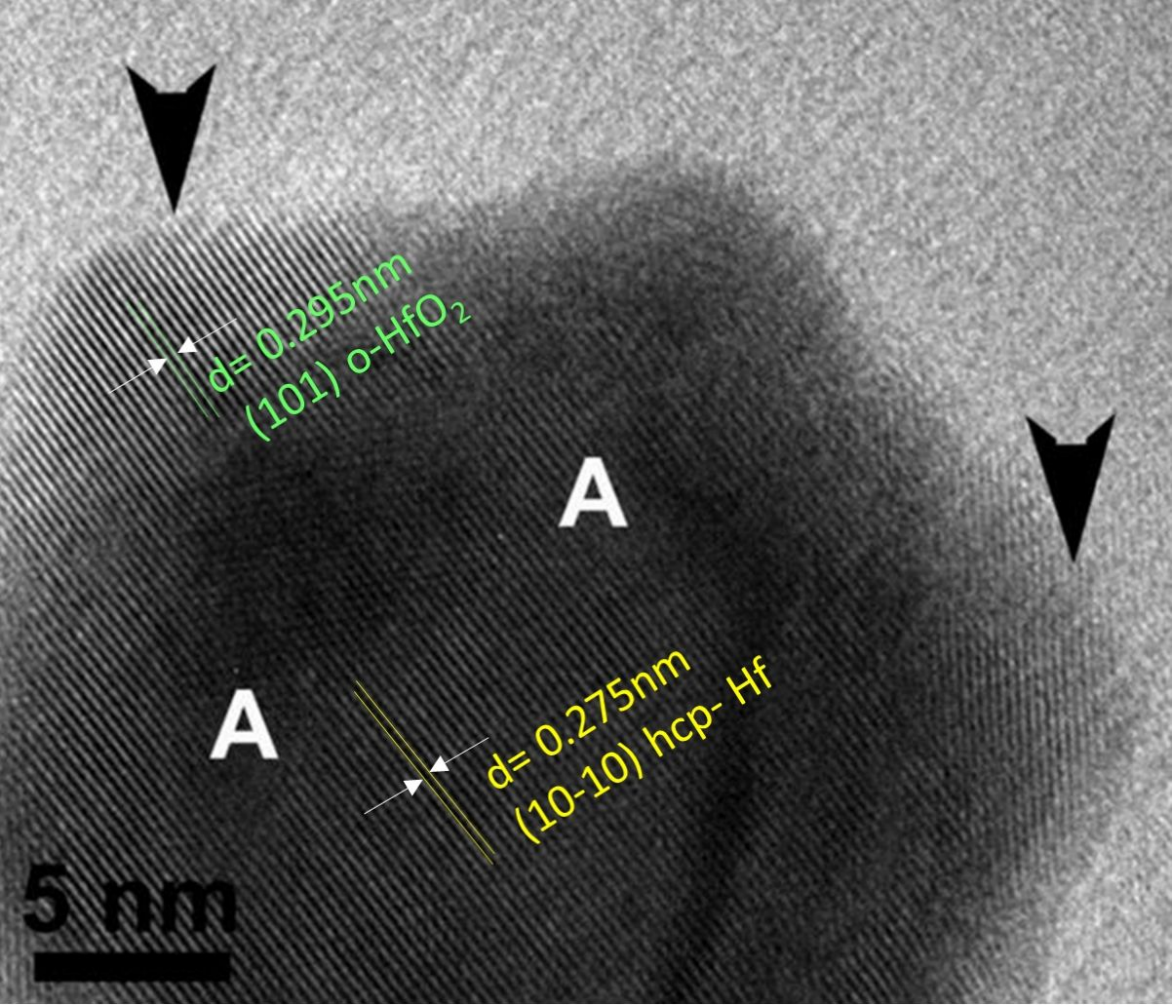
HRTEM image of a Hf nanoparticle showing a distinct core–shell structure: In the core area lattice fringes of *d* = 0.275 nm corresponding to (10−10) Hf can be seen marked as A. The shell is composed of orthorhombic HfO_2_ nanocrystallites indicated by arrows exhibiting lattice fringes of *d* = 0.295 nm corresponding to (101) HfO_2_ embedded in an amorphous layer.

Structural characterization of the nanoparticles was performed by high-resolution electron transmission microscopy (HRTEM) and X-ray diffraction. In both cases the nanoparticles were exposed to ambient air prior to characterization. From analysis of HRTEM images it is evident that Hf NPs have a distinct core–shell structure, consistent with a Hafnium core covered with Hafnium oxide (Figure 2). In the core the distance between adjacent planes is equal to *d* = 0.275 nm, value corresponding to the (10−10) crystal planes of hexagonal close-packed (hcp) Hf (Figure 2, regions A, JCPDS 00-038-1478). In the shell, the coexistence of nanocrystallites of orthorhombic HfO_2_ (Figure 2, regions with arrows) within amorphous regions is observed. The distance between adjacent planes in the shell is equal to *d* = 0.295 nm, which corresponds to (101) planes of orthorhombic crystalline HfO_2_ (JCPDS 00-048-1173). The formation of the oxide shell is due to oxidation when the Hf nanoparticles are exposed to ambient air. The scenario that the oxidation takes place inside the vacuum chamber is excluded, because of the distinct core–shell structure of the Hf NPs. If oxidation would take place inside the aggregation zone during NP formation, we would have hafnium oxide in the core. We also exclude the oxidation in the deposition chamber after landing of the nanoparticles on the substrate. Since we have already excluded oxidation in the aggregation zone, there is no physical reason why there should be oxidation within the deposition chamber where the pressure is lower than within the aggregation zone. The distinct core–shell structure is typical of most metal nanoparticles when exposed to air at room temperature [44–47] and it is well understood on the basis of the Cabrera–Mott model [48].

**Figure 3:**
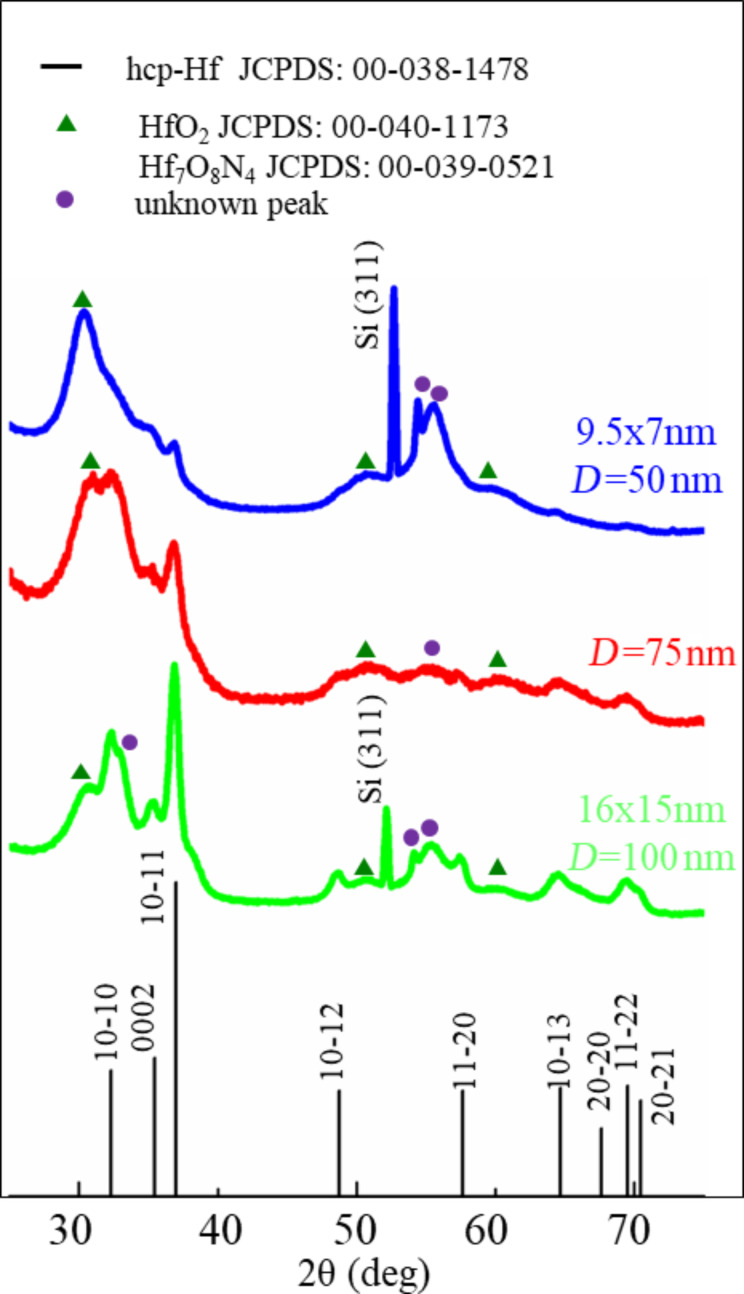
X-ray pattern of hafnium NPs on Si substrate synthesized for different aggregation-zone lengths *D* = 50, 75 and 100 nm.

The structure of Hf nanoparticles was further examined by grazing incidence X-ray diffraction (GIXRD). The NPs were prepared under the conditions described in the Experimental section for aggregation-zone lengths of *D* = 50, 75 and 100 mm. In all cases the majority of the peaks was matched with hexagonal close-packed (hcp) Hf (Figure 3). The hcp structure of the Hf NPs agrees with previous thermodynamic calculations presenting the phase diagram of Hf NPs with the particle size as function of the temperature [37] as we expect it in the vacuum system during NP growth. There are also some peaks that are due to a compound of Hf with oxygen (O) and/or nitrogen (N). This result is consistent with HRTEM measurements. The exact nature of the shell cannot be identified from the X-ray patterns, because the peaks of this crystalline phase match with several compounds of Hf with O and N, such as HfO_2_ or Hf_7_O_8_N_4_ (Figure 3). The peak broadening observed as nanoparticles size decreases, is consistent with the reduction of crystal grain size for smaller nanoparticles [49].

The ratio *I*_HfO2_/I_Hf_ of the intensities of HfO_2_ at 2θ = 30.8° and of (10−10) Hf at 2θ = 36.87° is increasing with decreasing nanoparticle size. This result is due to the enhanced oxidation of smaller nanoparticles due to their higher surface-to-volume ratio. Although broadening of diffraction peaks due to the sample arises from crystal size and the presence of strains and defects in the crystal [49–50], we made a simplified assumption that the X-ray peak broadening is due to the crystal size only, in order to estimate roughly the crystal grain size by applying the Scherrer equation (see Supporting Information File 1, Section 1) [49]. The size of the crystallites was calculated to be ca. 11 nm. This value is smaller than the average nanoparticle diameter defined from TEM images equal to *D* = 16 nm. This observation indicates that the nanoparticles are not single crystals, which is expected in our case, because the nanoparticles have a core–shell structure.

### Soft landing – NP shape

**Figure 4:**
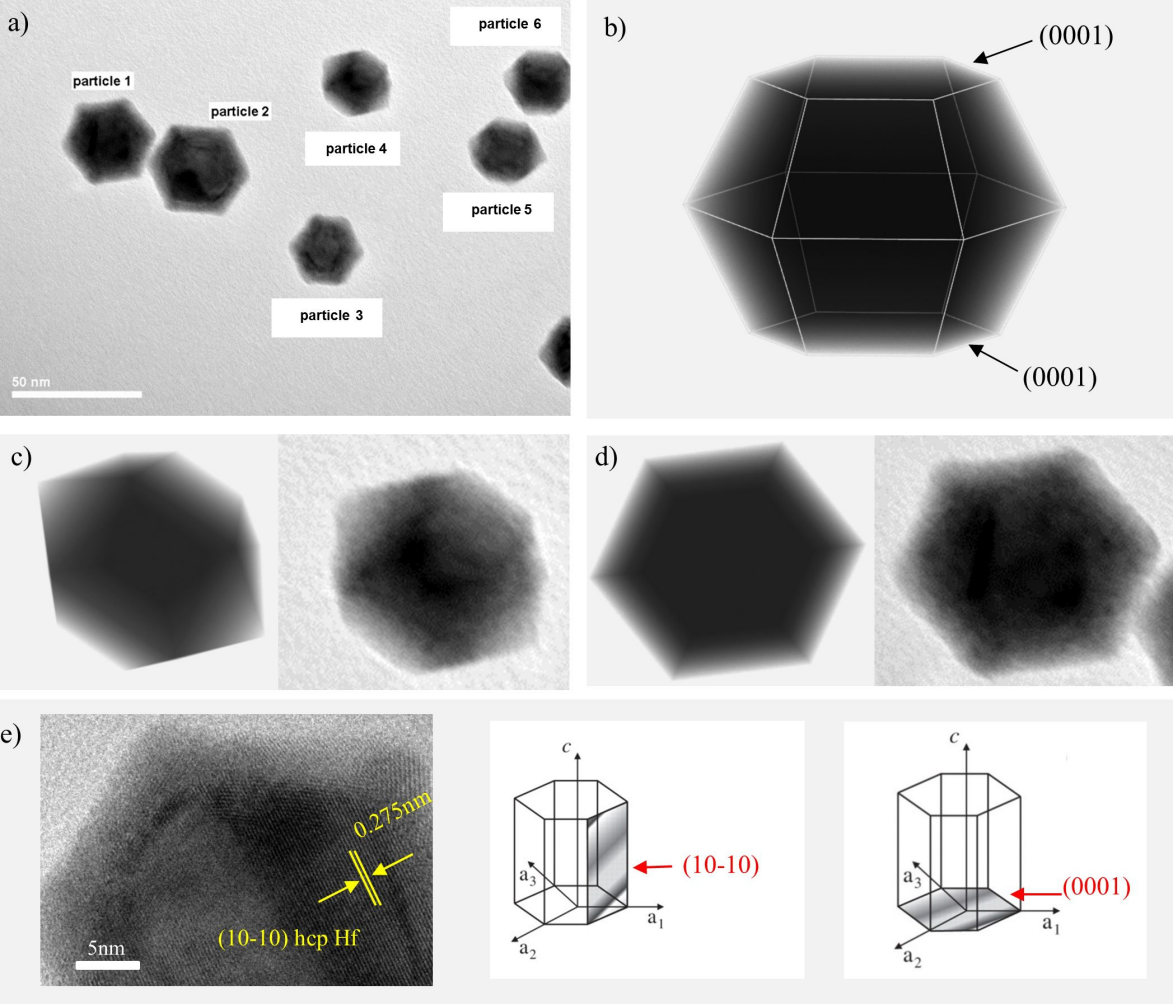
a) Bright-field TEM image of Hf nanoparticles; b) 3D geometric model of the truncated hexagonal biprism constructed with the technique of volume rendering; c) geometric model of the volume-rendered truncated biprism placed on one biprismatic plane (left) and the corresponding TEM of nanoparticle 4 (right); d) geometric model of the volume-rendered truncated biprism placed on the basal plane (left) and the corresponding TEM of nanoparticle 1 (right); e) HRTEM image of the basal plane of nanoparticle 1. Lattice fringe measurements correspond to (10−10) plane of hcp Hf, which is perpendicular to the (0001) plane. The schematic of the unit cell of the hcp crystal system shows that (10−10) and (0001) planes are perpendicular.

The shape of the nanoparticles cannot be solely defined from TEM images, because they give 2D projections of the 3D particle. Concerning Hf NPs, two different faceted shapes are observed (Figure 4a). The shape of the nanoparticles is usually determined by surface-energy minimization. Each free surface is characterized by its surface energy, the physical origin of which is the fact that atoms at the free surfaces have unsaturated chemical bonds. The crystalline solids exhibit different surface energies for the various crystalline planes, which leads to a deviation of the particle shape from the spherical shape [39]. The knowledge regarding the shape of hcp metals is very limited. Studies on hcp Ru nanocrystals revealed that they formed both truncated biprisms and hourglass shapes [51–52]. According to a recent publication of Luo et al., the equilibrium crystal shape of hcp metals takes the form of a truncated hexagonal biprism, with the basal plane corresponding to the (0001) plane, whereas the other biprismatic planes vary from one metal to the other [53].

In order to define the shape of hcp Hf NPs we constructed the geometric model of a truncated biprism through volume rendering (Figure 4a). Volume rendering techniques display 2D projections of a 3D model, by simulating the propagation of light rays into a semitransparent materials volume and creating the illusion of depth. Thus, similar to bright-field TEM images thin regions appear brighter than thick ones. In order to identify the shape of the nanoparticles we rotated the volume-rendered solid and checked if TEM images of the nanoparticles matched with the volume-rendered 2D projections. If the constructed structure of the truncated biprism is placed on its basal plane, the volume-rendered 2D projection matches perfectly with the TEM image of particles 1–3 (Figure 4). If the truncated biprism is placed on one of its biprismatic planes, the 2D projection coincides with the TEM image of particles 4–6 (Figure 4). Hence, we conclude that the observed shapes correspond to different orientations of one geometric model. The crystal plane of the basal plane of the Hf nanoparticles was identified (the basal plane is the plane shown on TEM images of nanoparticles 1–3 in Figure 4). HRTEM image and lattice spacing measurements of particle 1 reveal that the plane perpendicular to the basal plane correspond to (10−10) hcp Hf. Thus, the basal plane has to be (0001). This analysis provides evidence that the hcp Hf nanoparticles have the shape of truncated hexagonal biprisms with the basal plane being the (0001) plane. These results confirm the theoretical model proposed in a recent publication by Luo and co-workers [53].

### Energetic deposition (*V*_s_ ≠ 0 kV) – deposition conditions

During the energetic deposition, Hf NPs were produced by inert-gas condensation technique under exactly the same deposition conditions described above, except for the substrate bias voltage *V*_s_. These deposition conditions results in the vast majority of the NPs being charged (see Supporting Information File 1, Section 2). The energetic deposition of nanoparticles was studied for substrate bias values of *V*_s_ = 2 and 4.5 kV and for Hf NPs with the smallest mean dimensions (ca. 9.5 nm × 7 nm, aggregation-zone length *D* = 50 mm). The kinetic energy of the particles is governed by two terms, the pressure difference between the two chambers and the energy gained because of the applied electric field. Thus, the energy per atom equals *E*/*N* = *E*_0_/*N* + *eV*_s_/*N*. The term *E*_0_/*N* corresponds to the initial energy per atom due the pressure difference and is about 0.1 eV/atom for NPs produced by IGC [40] and *eV*_s_/*N* is the energy per atom gained by the electric field. Considering that the NP are approximately spherical, the number of atoms *N* composing a NP can be estimated by the following relation [54]: *N* = (*R*_N_/*R*_WS_)^3^ ≈ 17295 atoms/NP, where *R*_N_ is the mean radius of the nanoparticle, and *R*_ws_ is the Wigner–Seitz radius of an atom. If NPs are considered to be singly charged, the energy per atom *E*/*N* for *V*_s_ = 2 kV and *V*_s_ = 4.5 kV equals 0.22 eV/atom and 0.36 eV/atom, respectively. Since the energy values are one order of magnitude lower than the cohesive energy of Hf (6.31 eV/atom [55]), it is quite improbable that all clusters collide with each other to be completely fragmented. Even if a minority of multi-charged NPs (doubly/triply charged) exists and the maximum substrate voltage *V*_s_ = 4.5 kV is applied, the energy per atom will still be below 1 eV/atom, which is still below the cohesive energy of hafnium. Thus, from these energy values we expect mainly the deformation of the NPs [43].

### Energetic deposition – morphological characterization

**Figure 5:**
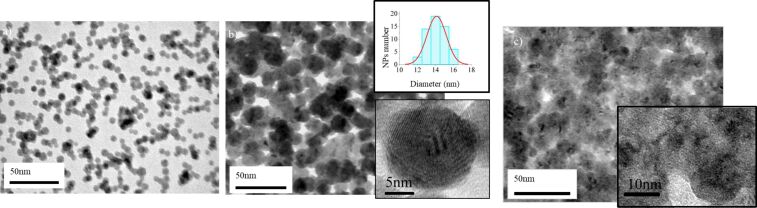
TEM showing the morphology of Hf NPs deposited at different substrate voltages *V*_s_: a) *V*_s_ = 0 kV; the mean diameter is 9.5 nm. b) *V*_s_ = 2 kV; the impact energy leads to nanoparticle flattening. The mean diameter of the flattened NPs is 14 nm. c) *V*_s_ = 4.5 kV; the increment of impact energy leads to further deformation of the nanoparticles.

TEM images of Hf nanoparticles deposited using substrate bias voltages of *V*_s_ = 0, 2 and 4.5 kV reveal that the morphology of the NPs is affected strongly by the applied substrate voltage *V*_s_ (Figure 5). For the case of *V*_s_ = 0 kV (soft-landing regime), Hf nanoparticles preserve their shape and composition (see previous section). When *V*_s_ = 2 kV, the nanoparticles retain their integrity but are plastically deformed. From the size distribution of the NPs diameter it seems that the NPs become flattened (Figure 5b). Their average diameter increases from 9.5 nm for *V*_s_ = 0 kV to ca. 14 nm for *V*_s_ = 2 kV. A similar effect where the applied voltage leads to flattening has been also observed for Ag nanoparticles [56]. When the substrate voltage is *V*_s_ = 4.5 kV, NPs get completely deformed and it cannot be excluded that they are partially fragmented. In this case the formed nanoparticle film barely consists of its initial “building blocks” (Figure 5c).

The morphology of the nanoparticles film during inert-gas condensation depends on the relation between the cohesive energy and the energy per atom *E*_at_ of the nanoparticle when landing on the substrate. The cohesive energy can be regarded as the required energy to separate the metallic crystal into individual atoms by destroying the metallic bonds [57]. During soft landing the energy per atom stays well below the cohesive energy of the nanoparticles and the nanoparticles preserve their composition [43,58]. During energetic deposition, plastic deformation occurs when the energy per atom is close to the cohesive energy of the nanoparticles. The degree of deformation depends on the impact energy. When the kinetic energy of the nanoparticles is further increased so that the *E*_at_ atom is slightly above the binding energy, deformation and partial fragmentation of the nanoparticles occur [40,43].

**Figure 6:**
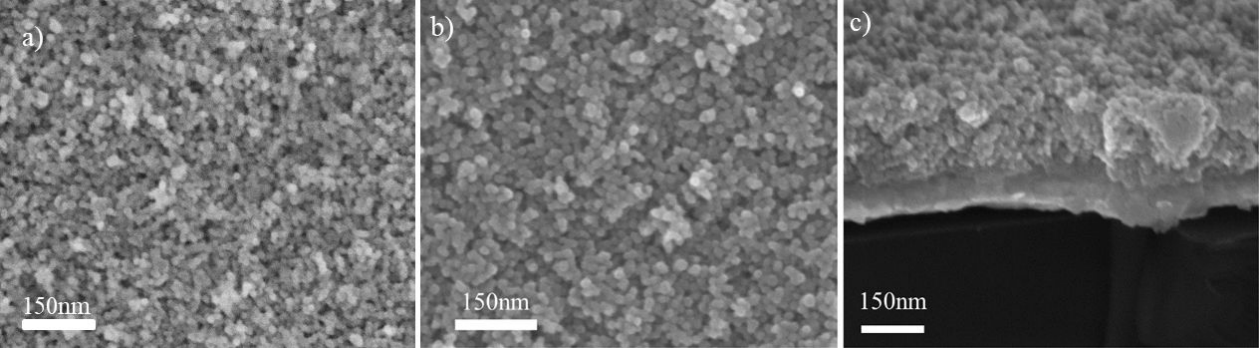
SEM images of Hf NPs film formed with energetic deposition and substrate bias: a) *V*_s_ = 2 kV; b,c) *V*_s_ = 4.5 kV, the porous structure of the film is revealed.

From SEM images of samples formed for longer deposition times we observed that porous granular composed of Hf nanoparticles are formed for all substrate voltage values (Figure 6). This is expected during soft landing, where pores are created due to the random stacking of nanoparticles on the substrate or on the lower nanoparticle layers [45,59]. In case of energetic nanoparticles deposition density and compactness of the film depend on the impact energy, which in our case remains low and results in the formation of porous films.

### Energetic deposition – structural characterization

**Figure 7:**
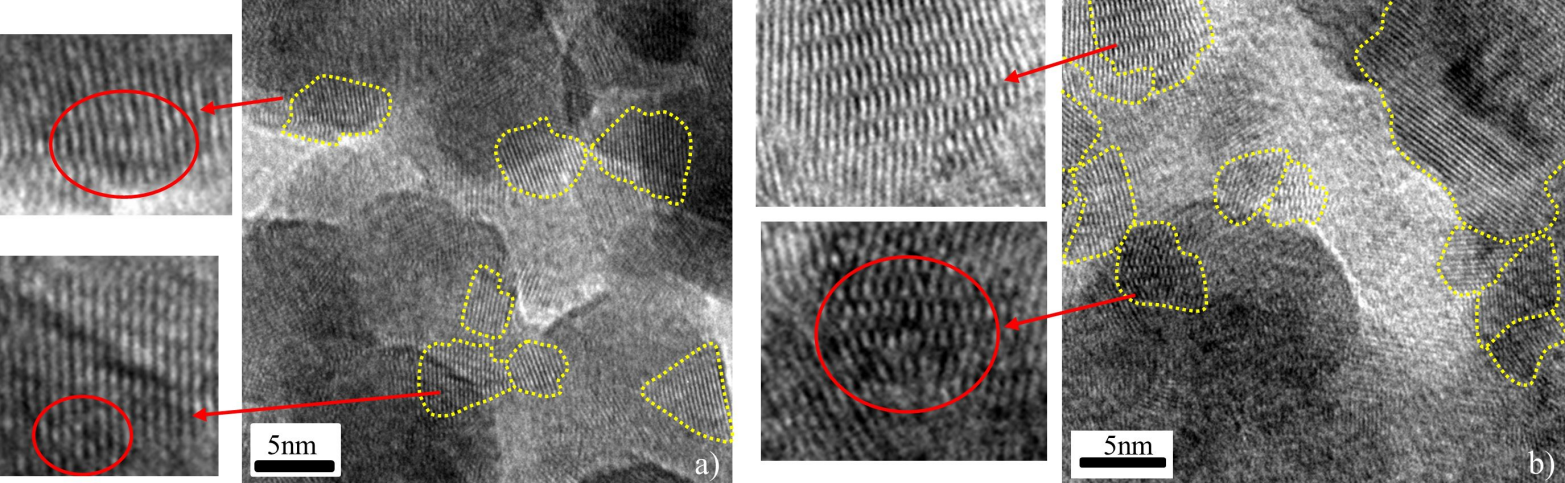
HRTEM characterization of Hf NTFs formed with energetic Hf NPs deposition and substrate bias: a) *V*_s_ = 2 kV and b) *V*_s_ = 4.5 kV. In both cases, the films are composed of nanosized crystal grains (yellow regions). A higher magnification of crystal grains shows distorted areas of their crystal lattice.

Structural characterization of the nanoparticles was performed by high-resolution electron transmission microscopy (HRTEM) and X-ray diffraction. In both cases the nanoparticles were exposed to atmospheric air prior to characterization. HRTEM images indicate that the NTFs formed for both cases of energetic deposition (*V*_s_ = 2 and 4.5 kV) are nanocrystalline. The individual crystallites are differently oriented, having dimensions below 10 nm and in many cases deformation of the crystal lattice is observed (Figure 7).

**Figure 8:**
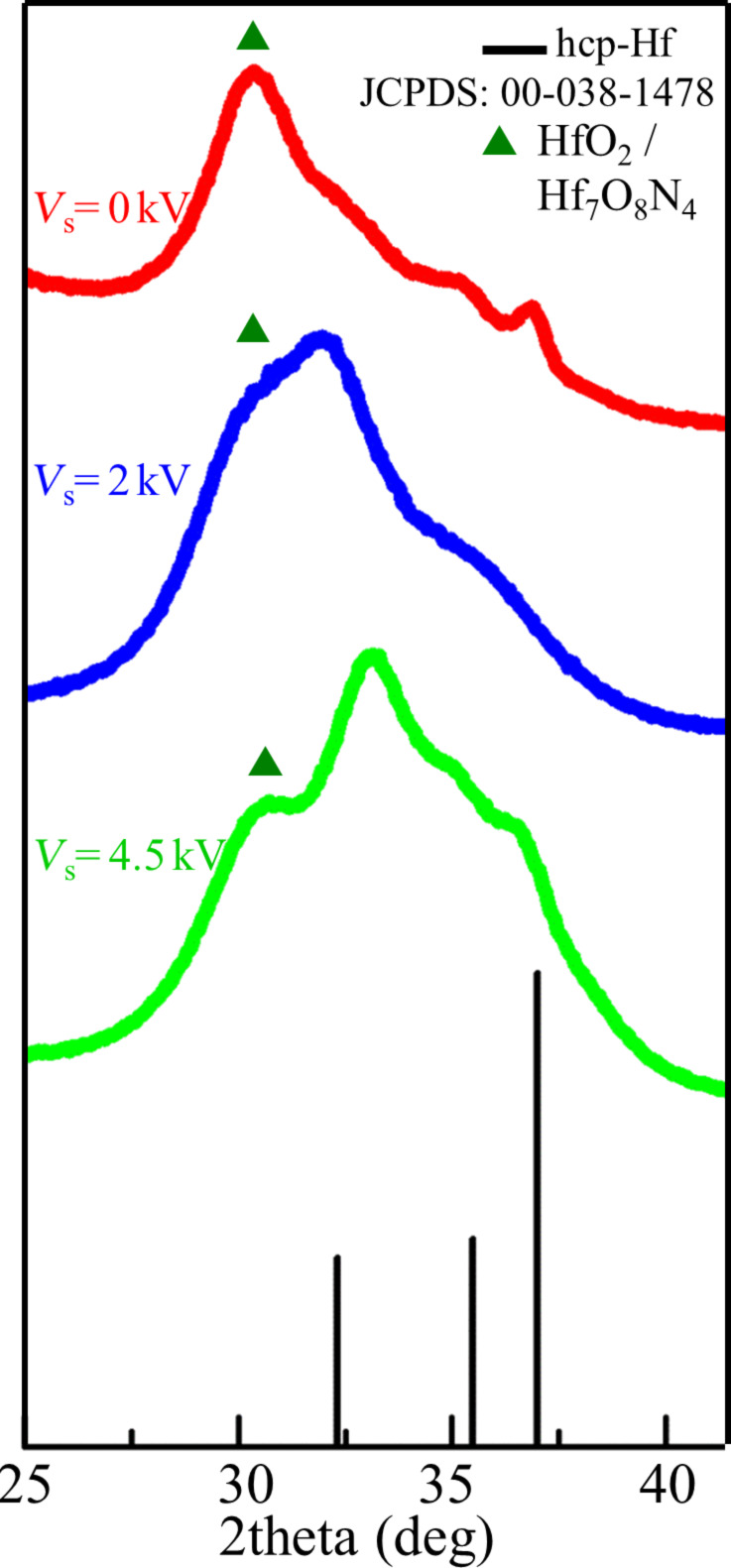
X-ray pattern of Hf NTFs deposited at different substrate voltages *V*_s_ = 0, 2 and 4.5 kV.

X-ray diffraction measurements show peaks matching with hexagonal close-packed Hf. There are also some unclear peaks that can be associated to compounds of Hf with oxygen and/or nitrogen, similar to ones observed in the soft-landing regime (Figure 8). The almost featureless and broad X-ray diffraction peaks are in accordance with the findings of the HRTEM (Figure 7). Both lattice imperfections and smallness of the crystal grain contribute to X-ray diffraction line broadening. Furthermore, when the grain size is reduced to a few nanometers the number of atoms located in the grain boundaries becomes comparable with the number of atom forming the nanocrystal [60–61]. Then the X-ray pattern is a superposition of diffractions from the crystal grains and scattering from grain boundaries, where a broad spectrum of interatomic distances exist. The resulting X-ray diffraction pattern appears very similar to that of an amorphous material.

Additionally, from the X-ray patterns, it is observed that the relative intensity of the peaks HfO_2_ and Hf *I*_HfO2_/*I*_Hf_ decreases with increasing the substrate voltage. This means that the oxidation of NTFs is reduced with substrate voltage. We attribute this observation to the pore volume of the different samples. The existence of pores in the prepared films allows for the partial oxidation of hafnium upon exposure to ambient air. Pore volume and surface depend on each other so that surface area decreases with decreasing pore volume. This means that oxidation is reduced in samples with low pore volume compared to samples with high pore volume. Based on this we expect a porosity decrement for samples prepared with higher substrate voltages.

### Nanomechanical properties and porosity of Hf NTFs

**Figure 9:**
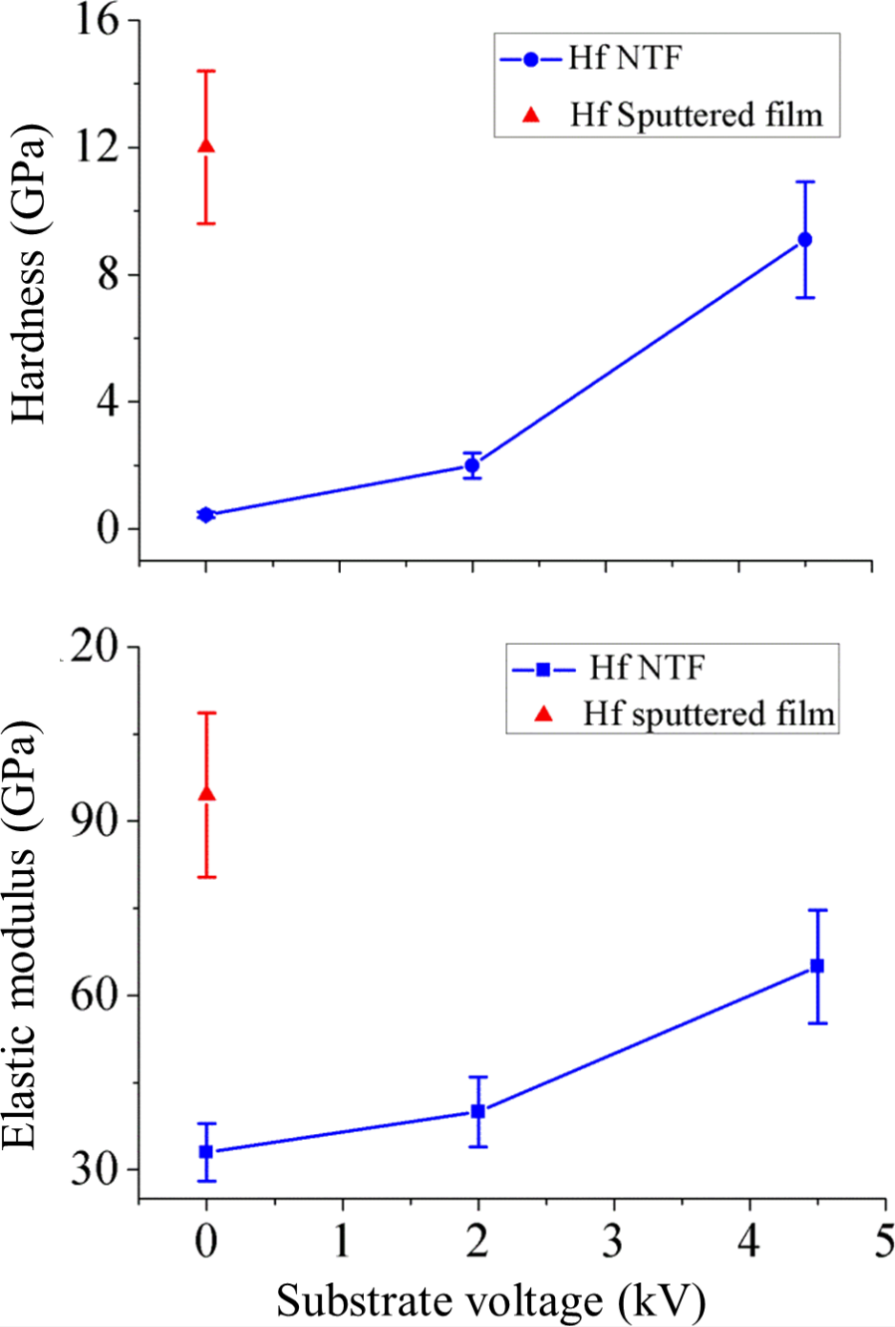
Dependence of hardness (*H*) and elastic modulus (*E*) of Hf NTFs as functions of the substrate voltage *V*_s_.

Hardness (*H*) and elastic modulus (*E*) of the nanocrystalline porous Hf NTFs were determined by nanoidentation measurements. (The load–displacement curves of Hf NTFs are presented in Supporting Information File 1, Section 3.) For comparison, the hardness and elastic modulus of a radio-frequency (RF)-sputtered 120 nm thick Hf film, was measured with the same technique as the NTFs. When *V*_s_ = 0 kV, elastic modulus and hardness are *E* = 33.6 GPa and *H* = 0.45 GPa, respectively. With increasing substrate voltage the mechanical properties are improved. For *V*_s_ = 2 kV, elastic modulus and hardness increase to *E* = 40.4 GPa and *H* = 2 GPa, respectively. For *V*_s_ = 4.5 kV, further increments to *E* = 65.8 GPa and *H* = 9.1 GPa are observed. These values tend to approach the mechanical properties of the RF-sputtered Hf film, which are *E* = 94.5 GPa and *H* = 12 GPa. The results are summarized in Figure 9. These results demonstrate that porous nanoparticles thin films with improved mechanical properties can be fabricated with energetic nanoparticle deposition without any post treatment. To our knowledge these combined values are comparable to those reported in the literature for NTFs undergone mechanical reinforcement with other techniques [29–30].

**Figure 10:**
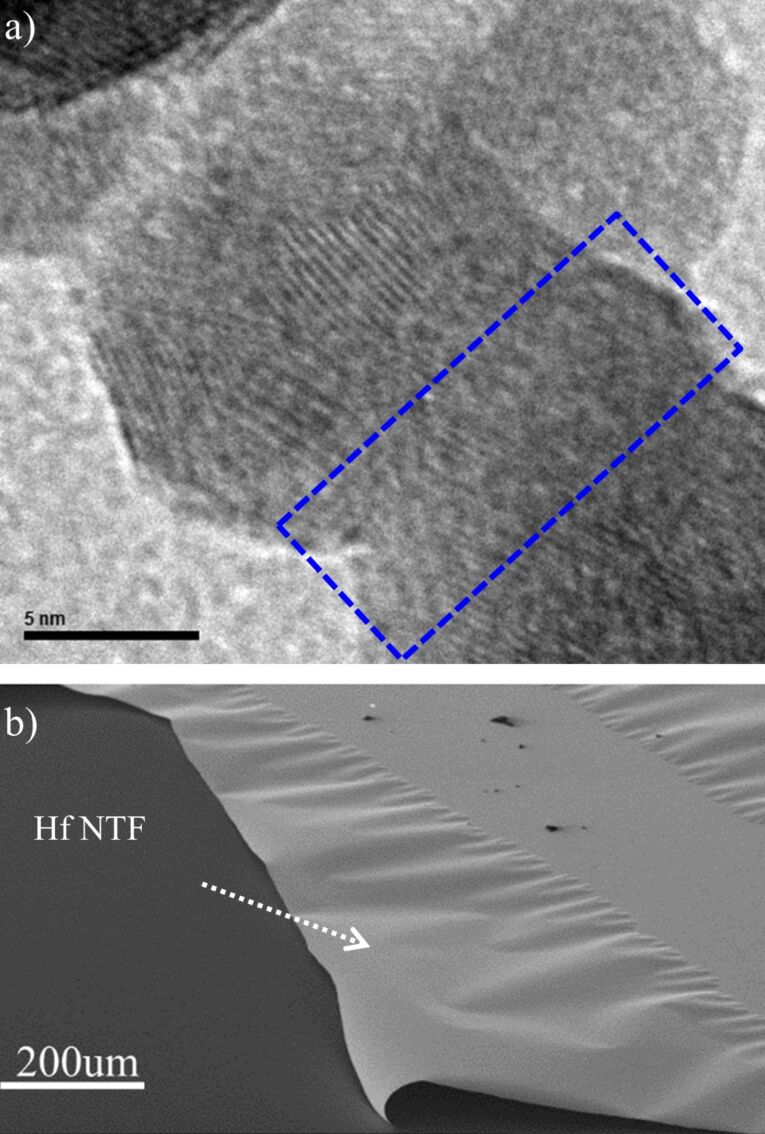
a) HRTEM image showing partial coalescence of Hf NPs deposited at a substrate bias of *V*_s_ = 2 kV; b) free standing Hf NTFs grown with *V*_s_ = 2 kV. The film, although consisting of nanoparticles, appears cohesive. The films thickness is ca. 120 nm.

We believe it is porosity and strength of the bonding between the grains that affect the mechanical properties of the films. In the case of *V*_s_ = 0 kV, fragile NP coatings with poor mechanical properties are formed due to the weak adhesive van der Waals interaction between NPs and substrate [62].When increasing the substrate voltage, the strengthening is attributed to the high temperatures that arise locally when the NPs impact on the substrate [63]. Due to the high temperatures lead to partial coalescence of the NPs and neck formation. HRTEM images support this estimation (Figure 10a).

### Porosity of Hf NTFs

The porosity of the NTFs formed for *V*_s_ = 0, 2 and 4.5 kV was estimated by applying the method of Ramakrishnan–Arunachalam (see Supporting Information File 1, Section 4) [64]. In this model the porous solid is considered a continuous medium with randomly distributed pores and the porosity *p* is the related to the mechanical properties of the film. The porosity of the NTFs can be tuned through the substrate voltage. A porosity decrement of the Hf NTFs is observed with increasing substrate voltage. During soft landing, a random stacking of the NPs leads to a very porous structure. When the substrate voltage is increased, partial coalescence (Figure 10a) of the NPs leads to more compact NTFs. The porosity values are 32%, 27% and 12% for *V*_s_ = 0, 2 and 4.5 kV, respectively. These results render energetic deposition of NPs as an alternative method to tune the porosity of the NTFs.

### 3D NTF patterning

**Figure 11:**
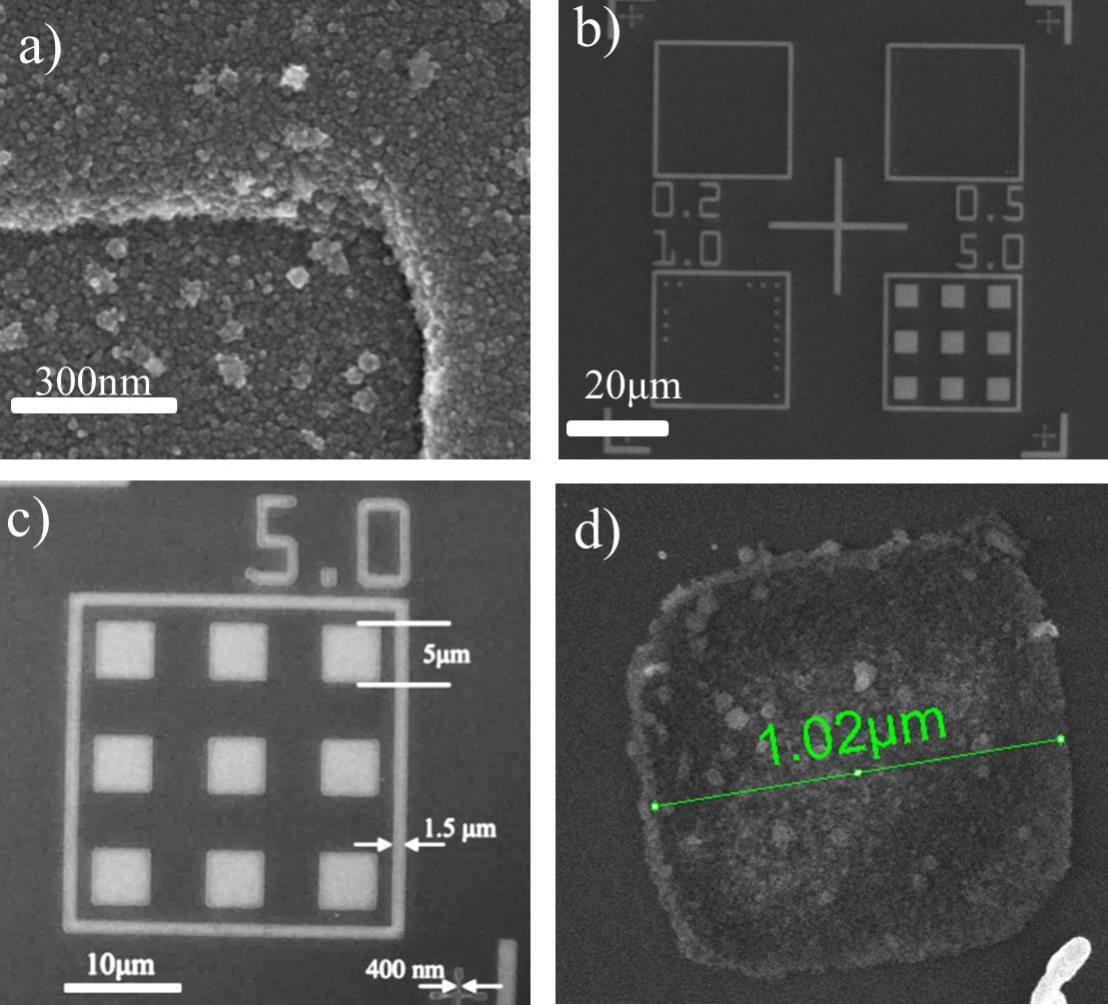
Scanning electron microscopy image of 3D patterns composed of Hf NPs deposited at a substrate voltage of *V*_s_ = 4.5 kV: a) The high-magnification image shows the granular structure of these patterns; b,c) the areas that appear white are structures formed from the nanoparticles. Large square structures of 5 μm size were successfully constructed. d) Cohesive structure with 1 μm side found at a random position on the substrate.

Cluster beam deposition combined with patterning for fabricating 3D objects has been successfully performed by other groups with techniques such as supersonic cluster beam deposition combined with stencil masks [65–66]. In our case we have successfully constructed 3D patterns of Hf NPs, exploiting energetic nanoparticle deposition. During deposition of the NPs the substrate bias was *V*_s_ = 4.5 kV. The height of the as-prepared structures was 70 nm. As apparent from SEM images (Figure 11) large square structures of 5 μm size were fabricated successfully, while there are only some remains of square structures of 1 μm size. It is likely that smaller structures were swept away during removal of the resist. However, the SEM images show that lines of widths down to 400 nm can be still obtained successfully with this technique. We did not try to construct other geometries and we did not test this method at other substrate bias values. But these results demonstrate that the method of energetic NPs deposition on patterned surfaces via lithography seems to have the technical potential to construct mechanically stable 3D patterns composed of nanoparticles.

## Conclusion

We have demonstrated the production and characterization of Hf nanoparticles and Hf nanoparticles thin films. A number of methods, including grazing X-ray diffraction, transmission electron microscopy, scanning electron microscopy, atomic force microscopy and nanoindentation measurements, were utilized for their characterization. Hf nanoparticles were fabricated by magnetron sputtering inert-gas condensation. They exhibit a hexagonal close-packed crystal structure and the shape of truncated hexagonal biprisms, and form core–shell structures upon exposure to ambient air. The hcp structure of the Hf NPs agrees with previous thermodynamic calculations presenting the phase diagram of Hf NPs as a function of particle size and temperature [37]. The shape of Hf NPs confirms the theoretical model proposed in a recent publication by Luo et al. for hcp nanocrystals [53]. Hf NTFs were grown by using energetic deposition. This technique allows the control of the kinetic energy of the charged NPs by applying a substrate bias voltage. The structural and nanomechanical properties of the nanoparticles thin films were investigated as a function of the substrate bias. The results reveal that in all cases porous NTFs of hexagonal close packed NPs are formed. The substrate bias affects their mechanical properties and their porosity. NTFs formed in the soft-landing regime exhibit poor mechanical properties. By increasing the kinetic energy of the nanoparticles, the mechanical properties of the NTFs were profoundly improved. We have estimated that Young modulus and hardness increases from 33.6 to 65.8 GPa and from 0.45 to 9.1 GPa, respectively, when the substrate bias is increased from 0 to 4.5 kV. Correspondingly , the porosity of the films changed from 32% to 12%. Furthermore, it was shown that NTFs can be patterned on the substrate in sub-micrometer dimensions using conventional lithography. We demonstrated the energetic deposition with Hf nanoparticles, but the technique can be applied to any charged metallic nanoparticles according to the required application. We propose this single-step method as an alternative solution to reinforce NTFs and to product mechanical stable porous NTFs.

## Experimental

### Deposition system

**Figure 12:**
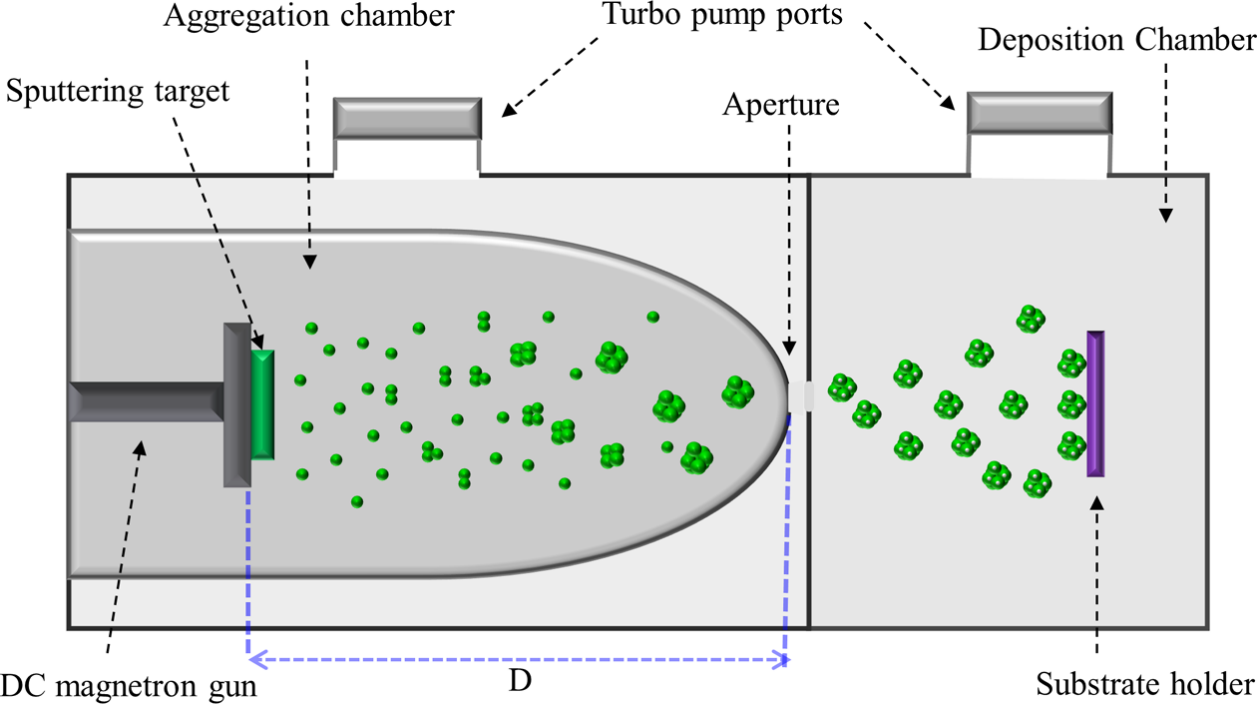
Schematic of the inert-gas condensation system used for nanoparticle growth.

Hf NPs were produced in a high-vacuum gas-phase nanoparticle deposition system (from Mantis Deposition Ltd., UK). A schematic representation of the system is shown in Figure 12. The system is composed of two vacuum chambers, the aggregation and the deposition chamber, which are connected via a small aperture (ca. 5 mm diameter). Pumping through the aperture leads to a pressure difference between the two chambers, with higher and lower pressure in the aggregation and deposition chamber, respectively. The nanoparticles are produced in the aggregation chamber, which contains a dc magnetron sputtering source. In the first stage of nanoparticle growth, atoms of the chosen material are produced by dc magnetron sputtering of a high-purity metallic target (99.95%). Due to collisions with Ar gas, cooling of the sputtered metal atoms takes place, leading to a supersaturated metal atom vapor. Collisions between the cooled metal atoms lead to nucleation and growth of initial clusters. Collisions between these initial cluster ‘seeds’ followed by coalescence lead to the growth of nanoparticles [67]. Due to the pressure gradient the nanoparticles enter the deposition chamber, where they land onto a suitable substrate. The nanoparticle size can be adjusted through the residence time in the aggregation zone. The residence time is affected by modifying the aggregation-zone length, which equals the distance *D* between the sputtering target and the exit aperture (Figure 12). The velocity of the nanoparticles is determined by their collisions with the carrier gas. Near the aperture a pressure drop of several orders of magnitude occurs over the length of several millimeters, which leads to a strong acceleration of the carrier gas due to expansion, with the gas atoms reaching a mean velocity that is close to the speed of sound. The nanoparticles embedded in the carrier gas will be accelerated due to collisions with the gas atoms. But the NPs cannot equilibrate their velocity with the gas atoms, because they have large mass and there is not enough time to reach equilibrium with the gas atoms. The lighter NPs reach higher velocities than the heavier NPs. But in all cases the NPs inside the deposition chamber have a kinetic energy below 0.1 eV/atom, the so-called soft-landing regime [68–70]. In general, the particle beam produced contains neutral and charged (positive and negative) NPs. The charging of NPs occurs in the plasma region where the electron and ion densities are high [40]. In our system it seems that the vast majority of Hf NPs is charged (see Supporting Information File 1, Section 2). There is a basic assumption that NPs are singly charged, although it cannot be excluded that a minority of multi-charged NPs exists [42,71].

The charge allows their electrostatic manipulation by simply biasing the substrate. The charged nanoparticles are accelerated by the electric field towards the substrate and the energy of landing can be controlled by the voltage applied on the substrate (*V*_s_). Thus the charged nanoparticles can either soft-land on the substrate (*V*_s_ = 0 kV) or collide with a high energy (*V*_sub_ ≠ 0 kV). Depending on the impact energy, several scenarios, such as derformation, fragmentation and implantation of the NPs, are possible [40,43]. In this study we observed partial and complete deformation of the NPs.

### 3D NTF patterning

In order to fabricate 3D NTF patterns, a spin-coated PMMA film on a Si substrate was initially patterned via e-beam lithography with square structures with side dimensions ranging from 200 nm to 5 μm. Then Hf nanoparticles were deposited on the patterned surface at a voltage *V*_s_ = 4.5 kV. Finally, after the deposition the resist was removed using Piranha solution, because it could not be removed with acetone. This observation suggests that the polymer matrix has undergone a change during energetic deposition of nanoparticles. A possible explanation is that the clusters locally heat the PMMA above glass transition temperature, which results in their immersion into the polymer matrix forming a metal–polymer composite subsurface layer. This phenomena of has been observed by the groups of Milani and Popok [72–73].

### NP characterization

Structural characterization of the NTFs was performed by using grazing incidence X-ray diffraction (GIXRD) and high-resolution transmission electron microscopy. The X-ray measurements were performed on a Panalytical X’Pert Pro Diffractometer, using Cu Kα radiation at room temperature. The instrument was operated in a continuous-scan mode with increments of 0.05°, and the counts were accumulated for 45 s at each step. Identification of peaks was performed using the JCPDS database. All NTFs characterized with X-ray diffraction had a thickness of ca. 120 nm. Structural studies with TEM were conducted ex situ, after growth by using an FEI CM20 TEM operating at 200 kV. For TEM analysis the nanoparticles were deposited on carbon-coated Cu grids. Morphological characterizations of the NPs and NTFs were performed by field-emission scanning electron microscopy (FESEM FEI Nova Nano SEM 230) and atomic force microscopy (Veeco diInnova) in tapping mode.

The nanomechanical properties of the NTFs were determined by nanoindentation. Nanoidentation testing was performed with a Hysitron TriboLab Nanomechanical Test Instrument, which allows the application of loads from 1 to 30.000 μN and records the displacement as a function of the applied loads with high resolution of load (1 nN) and displacement (0.04 nm). In all tests, ten indents with a spacing of 50 μm were averaged. Nanoindentation tests were performed using the displacement-control protocol. The durations of loading and unloading are 20 s, and the holding time at the maximum load is 2 s. All nanoindentation measurements have been performed, with a standard three-sided pyramidal Berkovich probe. Hardness (*H*) and elastic modulus (*E*) values were extracted from the experimental data (load–displacement curves) using the Oliver–Pharr method [74].

## Supporting Information

The Supporting Information File 1 provides some details about:

a) The Hf crystallite size estimation with Scherrer equation.

b) The experiment for testing the charge of Hf NPs.

c) The nanoidentation load-unload curves of the Hf NTFs.

d) The application of the method of Ramakrishnan–Arunachalam in order to estimate the porosity of the Hf NTFs.

File 1 Additional experimental data.
