# Supplementary material for: Synthesis of hafnium nanoparticles and hafnium nanoparticle films by gas condensation and energetic deposition
Source: Beilstein J Nanotechnol. 2018 Jun 27;9:1868–80. doi: 10.3762/bjnano.9.179 (PMC6036986; doi:10.3762/bjnano.9.179)
Supplement: File 1 — Additional experimental data. [file Beilstein_J_Nanotechnol-09-1868-s001.pdf]

Supporting Information

for

Synthesis of hafnium nanoparticles and hafnium nanoparticle films by gas condensation and energetic deposition

Irini Michelakaki*[1], Nikos Boukos[2], Dimitrios A. Dragatogiannis[3], Spyros Stathopoulos[1], Costas A. Charitidis[3] and Dimitris Tsoukalas[1]

Address: [1]Department of Physics, National Technical University of Athens, Heroon Politechniou 9, Zographou - Athens, 15780, Greece; [2]Institute of Nanosciences and Nanotechnology, National Centre for Scientific Research Demokritos, Patriarchou Grigoriou E' & Neapoleos Str., Aghia Paraskevi - Athens, 15310, Greece and [3]School of Chemical Engineering, National Technical University of Athens, Heroon Politechniou 9, Zographou - Athens, 15780, Greece

Email: Irini Michelakaki* - michelakaki.irini@gmail.com

* Corresponding Author

Additional experimental data

1 Scherrer equation

Scherrer equation was used in order to estimate the crystallite grain size:

$$L = \frac{0.9\lambda}{\beta_D * \cos\theta},$$

where L is crystal grain size, $\lambda = 1.5418$ Å is the wavelength of Cu Kα radiation, and β_D is the FWHM of the peak at angle 2θ.

In order to calculate the FWHM, we used the X-ray pattern of the biggest Hf NPs (16 nm × 15 nm), which has more intense and narrow peaks (Figure 3). The FWHM was estimated from fitting the X-ray peaks with a pseudo-Voigt function. For the Hf crystal diameter determination we used the most intense and narrow (10−10) Hf peak with $\beta_D = 0.73°$ and the crystal grain size was calculated equal to $L = 11$ nm.

2 Experiment for testing the charge of Hf NPs

We have tested the charge of Hf nanoparticles with the following experiment (Figure S1): Initially, we recorded the deposition rate on the substrate using a voltage $V_s = 0$ V, then gradually we increased the applied voltage while simultaneously recording the deposition rate. From the data it appears that increasing the applied voltage leads to a reduction of the recorded deposition rate, regardless of the sign of the applied voltage. From these data, it appears that the vast majority of the produced nanoparticles are charged, either positively or negatively, since the trajectory of the nanoparticles is affected when voltage is applied. The applied voltage, depending on the polarity, leads to attraction of clusters of one sign and deflection of clusters with opposite sign. As voltage is increased, the negatively charged NPs beam is more and more focused towards the sample holder, and thus not monitored by the quartz crystal monitor (QCM). At the same time the positively charged nanoparticles beam is deflected by the sample holder and thus not reaching the QCM (Figure S1b).

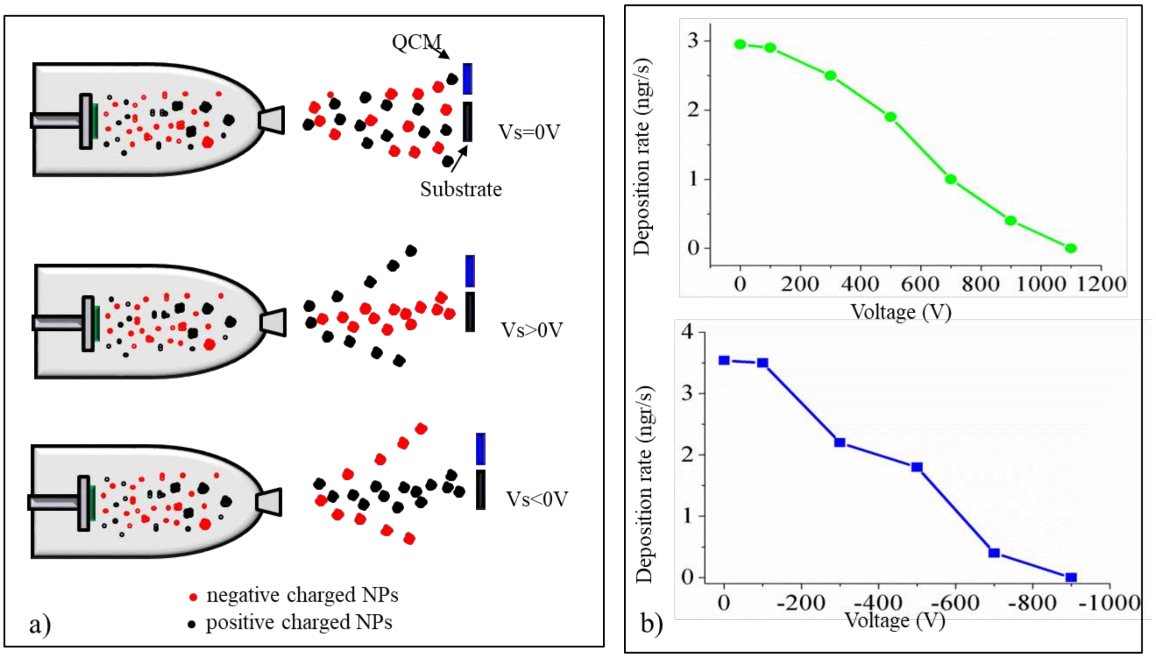

Figure S1: a) Schematic illustration of the nanoparticles beam when substrate voltages of different polarity are applied. b) Change of the recorded deposition rate of Hf NPs versus substrate voltage confirms that the majority of the nanoparticles are charged. When the applied voltage is positive (or negative), the negatively (positively) charged NPs beam is attracted towards the substrate, and the positively (negatively) charged beam is deflected from the substrate and thus the number of NPs that land on the quartz crystal monitor decreases, which manifests itself as a rate drop.

3 Nanomechanical properties

The nanomechanical properties of the NTFs were determined by nanoindentation. Nanoidentation testing was performed with a Hysitron TriboLab Nanomechanical Test Instrument, which allows the application of loads from 1 to 30.000 μN and records the displacement as a function of applied loads with high resolutions of load (1 nN) and displacement (0.04 nm). In all tests, ten indents with a spacing of 50 μm were averaged. Nanoindentation tests were performed using the displacement-control protocol. The durations of

loading and unloading are 20 s, and the holding time at the maximum load is 2 s. All nanoindentation measurements have been performed, with a standard three-sided pyramidal Berkovich probe. Hardness (*H*) and elastic modulus (*E*) values were extracted from the experimental data (load–displacement curves) using the Oliver–Pharr method.

Nanoidentation load–displacement curves

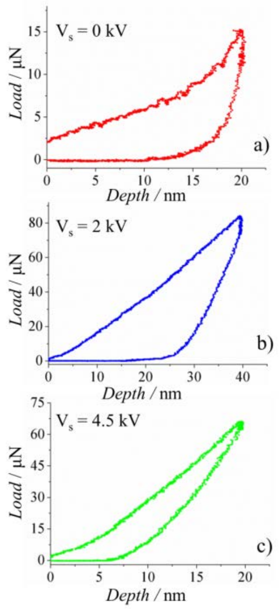

Figure S2: Nanoidentation load–displacement curves of ca. 120 nm Hf nanoparticle thin films deposited on Si substrate at substrate voltages of a) V_s = 0 kV, b) V_s = 2 kV and c) V_s = 4.5 kV.

4 Method of Ramakrishnan–Arunachalam

In this method the porous solid is considered a continuous medium with randomly distributed pores and the effect of porosity *p* on the elastic modulus *E* is described through the equation:

$$E_0 = \frac{E_p \left(1 + b_E \, p\right)}{\left(1 - p\right)^2},$$

where E_0 is the elastic modulus of the solid without pores, E_p is the elastic modulus of the porous solid, $b_E = 2–3\nu_0$ is a quantity that depends on the Poisson ratio of the solid ν_0 without pores. For determination of the elastic modulus of the Hf film without pores we have prepared a hafnium film with thickness of 120 nm on $SiO_{2,}$ using radio-frequency (RF) magnetron sputtering. The elastic modulus of this film was determined through nanoidentation and was found to be E = 94.5 GPa. The load–displacement curve of the RF-sputtered Hf film is shown in Figure S3

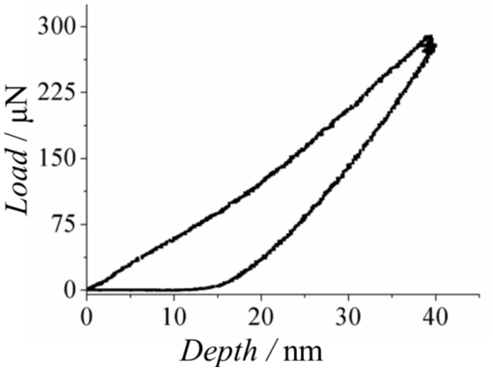

Figure S3: Nanoidentation load–displacement curve of a ca. 120 nm RF-sputtered Hf thin film on a Si substrate.
